# Need for discriminating between diagnostic and screening efficacy to estimate a biomarker based on case control and cohort studies

**DOI:** 10.1038/s41598-021-01904-0

**Published:** 2021-11-17

**Authors:** Liu Hui

**Affiliations:** College of Medical Laboratory, Dalian Medical University, Dalian, 116044 People’s Republic of China

**Keywords:** Diagnostic markers, Predictive markers

## Abstract

This study proposes the comprehensive index of biomarker (CIB), based on the consistency of a biomarker in case control (Youden index, J) and cohort studies (Crc), to evaluate biomarker efficacy. CIB was calculated as the mean of J and Crc. Analysis of the effect of sensitivity and specificity on CIB and ROC analysis of CIB were performed in simulated and actual datasets. J and CIB had similar values for high-probability events (say probability was 0.50), but there was a significant difference between J and CIB for low-probability events (say probability was 0.05). Therefore, as the subjects considered for diagnosis are usually symptomatic, the occurrence of a disease can be assumed to be a high-probability event. In contrast, as the subjects considered in screening for a disease are usually healthy and asymptomatic, the occurrence of a disease is assumed to be a low-probability event. Although J is the common index used to evaluate the diagnostic effectiveness, unfortunately, the J value is significantly larger than CIB value in a low-probability event, showing overestimation for screening purpose. CIB could have more potential than J for determining the screening efficacy of a biomarker. The efficacy of a biomarker could differ for diagnostic, screening, predictive, and prognostic purposes, and it would be better to evaluate the efficacy of biomarkers for specific systems or contexts.

## Introduction

One of the main purposes of identifying biomarkers is the diagnosis of diseases. In case–control studies, the potential relationship between a biomarker and the disease is examined by comparing the frequencies of the biomarker in diseased and non-diseased subjects, and the efficacy of a biomarker is usually described in terms of change in consistency, which is indicated by the Youden index (J)^1–3^. In a cohort study, a suspected biomarker is considered as an exposure factor, and exposed and unexposed subjects are observed until they develop the disease. The difference in the disease’s incidence between an exposed and non-exposed group, which is referred to as the consistency rate in cohort studies (Crc), indicates the role of the observed factor in the disease’s pathogenesis^4–6^. This type of research design is chronologically consistent in that the biomarker is the starting point for the diagnosis of the disease; therefore, a cohort study is probably more apt for identifying and analyzing biomarkers^7–9^. However, most studies that identify biomarkers use a case–control design rather than a cohort design^1,2^.

The relationship between the results of a case–control study and a cohort study is represented by the following formula^10^.

$$PPV = 1 - \frac{(1 - Spe)*(1 - m)}{{(1 - Spe)*(1 - m) + Sen*m}};\quad NPV = \frac{m*(1 - Sen)}{{Spe*(1 - m) - Sen*m}}$$

1 $$Crc = PPV - NPV$$

PPV and NPV represent the disease’s incidence (the frequency with which disease occurs) in the exposed and non-exposed (biomarker) group, respectively; Sen and Spe represent the positive rates of the biomarker in the disease group and the negative rates of the biomarker in the control group, respectively, in the case–control study; “m” represents the incidence in the total population; and Crc represents the consistency rate in the cohort study, which is differences in incidence between the two groups and also mean probability of incidence for a biomarker^11^.

The results of a case–control study and a cohort study are not always parallel. For example, if the occurrence probability of a biomarker is assumed to be 0.85 in the disease group and 0.05 in the control group, then the J value would be 0.80 (0.85–0.05) and the Crc would be 0.145 (m=0.01). When the cardinal number, that is, the probability in the control group, is relatively large, for example, 0.90 in the disease group versus 0.10 in the control group, then J is 0.8 and Crc is 0.082. This means that in case of a low-probability event (for example, m = 0.01), the difference between J and Crc would be significant. The occurrence of a disease is a low-probability event; therefore, J would be significantly larger than Crc. This indicates that the overestimation of J in case–control studies is a serious problem in determining the efficacy of a biomarker.

In the present study, I propose a comprehensive index for biomarkers, namely, CIB, that is based on a combination of consistency determined through both case–control and cohort studies, that is, J and Crc. CIB could overcome the limitations of J in low-probability events and have potential for determining the diagnostic efficacy of a biomarker and the difference between its diagnostic efficacy and screening efficacy.

## Materials and methods

### Calculation of CIB

The principle of the current analysis is to comprehensively evaluate the consistency of a biomarker in a case–control study and a cohort study in order to determine its efficacy. The efficacy of a biomarker is normally described in terms of J, which is the sum of the positive rates of a biomarker in the disease group (referred to as sensitivity or Sen) and the negative rates of the biomarker in the control group (referred to as specificity or Spe) minus 1^3^.

$${\text{J}} = {\text{Sen}} + {\text{Spe}} - 1$$

The consistency in a cohort study (Crc) is the sum of the incidence in the exposure group (positive group for a biomarker) (PPV) and the non-diseased rate (percentage of healthy individuals) in the non-exposure group (negative group for a biomarker) (NPV) minus 1 as follows^11^:

$${\text{Crc}} = {\text{PPV}} - ({\text{NPV}} - 1) - 1 = {\text{PPV}} - {\text{NPV}}$$

Using J and Crc, CIB is calculated as follows.

2 $${\text{CIB}} = \left( {{\text{J}} + {\text{Crc}}} \right)/2$$

In fact, CIB comprehensively incorporates Sen, Spe, PPV, and NPV.

When evaluating the diagnostic efficacy of a biomarker, its incidence in the total population (m) is assumed to be 0.50 because patients are typically symptomatic. For evaluating screening efficacy, including predictive power, the incidence (m) is assumed to be 0.05, because the subjects are usually healthy individuals without any symptoms. Thus, the range of CIB is 0–1, with a greater CIB value implying stronger predictive power of the biomarker.

### Evaluation of data from a case–control study

The basic principle of the analysis is to determine whether J can accurately reflect CIB.

Evaluation of J in a case–control study based on CIB calculated from both the case–control study and the cohort study was performed using Eq. (1) (which represents a definite relationship between the outcomes of a case–control study and a cohort study) and Eq. (2). The data for the test set were generated based on J, with large and small cardinal numbers in the control group and CIB calculated as shown in Table 1. The data in Table 1 show that the incidence of the disease influences the relationship between J and CIB. When the incidence is 0.50, the value of J is similar to (but not equal to) that of CIB. Therefore, in the case of a high-probability event (probability = 0.50), the efficacy of a biomarker can be described in terms of J. However, there was a significant difference between J and CIB in the case of a low-probability event (probability = 0.05).

**Table 1:** Relationship of comprehensive index of biomarker (CIB) with Youden’s index (J) according to incidence in the total population.

Cardinal number	Disease (%)	Control (%)	Incidence = 5%		Incidence = 50%	
			J	CIB	J	CIB
Large cardinal number in the control group	50	50	0.000	0.000	**0.000**	**0.000**
	55	45	0.100	0.060	**0.100**	**0.100**
	60	40	0.200	0.120	**0.200**	**0.200**
	65	35	0.300	0.181	**0.300**	**0.300**
	70	30	0.400	0.244	**0.400**	**0.400**
	75	25	0.500	0.310	**0.500**	**0.500**
	80	20	0.600	0.380	**0.600**	**0.600**
	85	15	0.700	0.460	**0.700**	**0.700**
	90	10	0.800	0.558	**0.800**	**0.800**
	95	5	0.900	0.699	**0.900**	**0.900**
	100	0	1.000	1.000	**1.000**	**1.000**
	5	5	0.000	0.000	**0.000**	**0.000**
	15	5	0.100	0.096	**0.100**	**0.189**
Small cardinal number in the control group	25	5	0.200	0.184	**0.200**	**0.296**
	35	5	0.300	0.267	**0.300**	**0.384**
	45	5	0.400	0.346	**0.400**	**0.467**
	55	5	0.500	0.421	**0.500**	**0.548**
	65	5	0.600	0.494	**0.600**	**0.630**
	75	5	0.700	0.564	**0.700**	**0.715**
	85	5	0.800	0.632	**0.800**	**0.804**
	95	5	0.900	0.699	**0.900**	**0.900**
	100	0	1.000	1.000	**1.000**	**1.000**

Boldface: Numerical value is similar.

Cardinal number: frequency in the control group.

### Evaluation of sensitivity and specificity

In case–control studies, biomarkers are assessed in already diseased individuals, and the power of a biomarker is typically expressed as the positive rates of the biomarker in the disease group (Sen) and the negative rates of the biomarker in the control group (Spe)^3^. As explained in the previous subsection, the diagnostic power of J may differ from that of CIB in the case of low-probability events. In this analysis, we examined whether Sen or Spe is more relevant with regard to CIB for biomarkers with the same J value for low-probability events. Evaluation of Sen and Spe in a case–control study based on CIB values showed that the J value differed for different Sen and Spe values. A scatter diagram was plotted with J on the X-axis and CIB on the Y-axis.

### Receiver operating characteristic analysis of CIB

Receiver operating characteristic (ROC) analysis is a common method used to evaluate the effectiveness of a diagnosis made using a biomarker^12,13^. The present study is to determine whether the ROC analysis was still available or not with using CIB instead of J.

**Figure 1:**
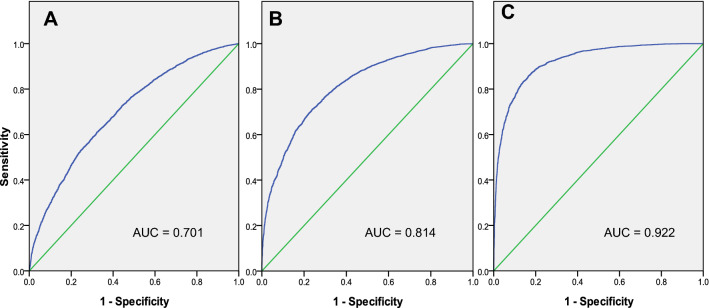
Receiver operating characteristic (ROC) analysis of simulated data in Model A (**A**), Model B (**B**) and Model C (**C**).

A model comprising four sets of simulated data was established. Four sets of normally distributed random numbers (100 ± 20, n = 5000; 115 ± 20, n = 5000; 125 ± 20, n = 5000; 140 ± 20, n = 5000) were generated using the SPSS statistical software (IBM Corp., Armonk, NY, USA). Model A consisted of the datasets 100 ± 20 and 115 ± 20; Model B consisted of the datasets 100 ± 20 and 125 ± 20; and Model C consisted of the datasets 100 ± 20 and 140 ± 20. ROC analysis was performed as shown in Fig. 1.

When the cardinal number (frequency in the control group) is relatively small (and Spe is higher), Crc could be infinity (Crc = 1). Therefore, if the frequency of a biomarker is less than 0.05 in the control group, it should be assigned a value of 0.05.

### Efficacy of CIB based on an actual dataset

Our previous research found that the tumor marker index (TMI) calculated from serial tumor markers can be considered as a simple tool for the diagnosis of gastric cancer^1^, so these results were considered to be apt for comparing the diagnostic and screening efficacy of J and CIB.

## Results

**Figure 2:**
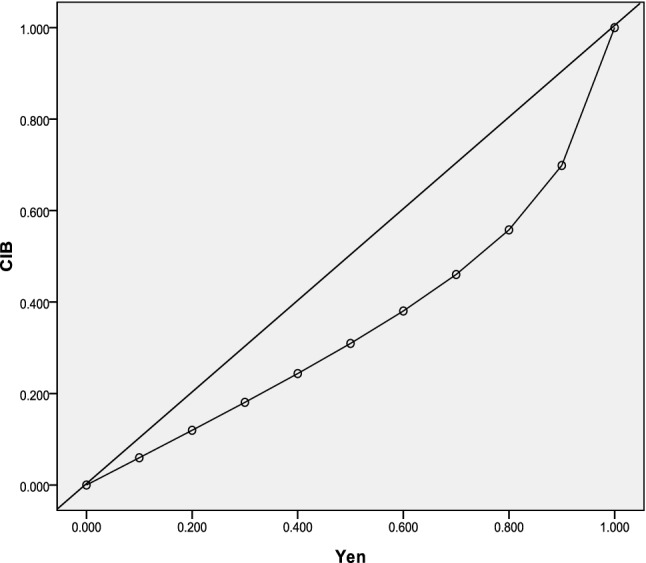
Relationship between Youden index (J) and comprehensive index of biomarker (CIB) (incidence = 0.05). CIB showed an unsteady increase with J for low-probability events.

The relationship between J and CIB is shown in Fig. 2. A plotted scatter diagram revealed that when the CIB level was 0.90, CIB was only 0.70 for an incidence rate of 0.05 in the total population.

**Figure 3:**
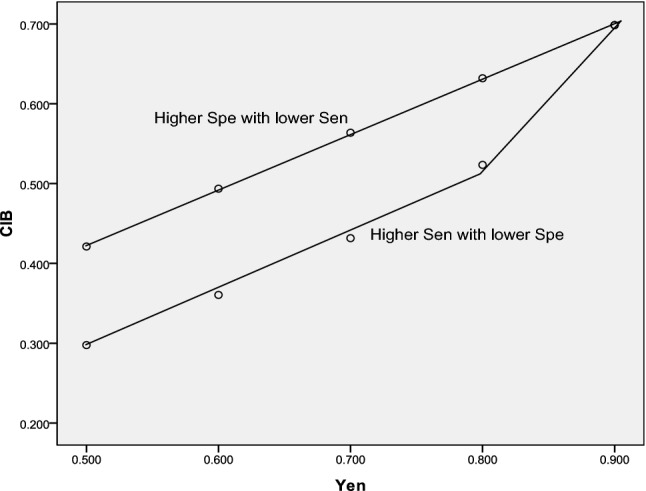
Relationship between comprehensive index of biomarker (CIB) and Youden index (J) for different sensitivity (Sen) and specificity (Spe) values (incidence = 0.05). There was a difference in CIB between biomarkers with the same J that had higher Spe and higher Sen.

The Sen and Spe of biomarkers in a case–control study were evaluated based on the CIB values, as shown in Fig. 3 and Table 2. There was a significant difference in J for different Sen and Spe values and CIB for a low-probability event (m = 0.05). As shown in Table 2, higher Spe (or a lower false-positive rate) could indicate better power of CIB for biomarkers with the same J.

**Table 2:** Evaluation of sensitivity (Sen) and specificity (Spe) in a case–control study based on comprehensive index of biomarker (CIB) (Incidence = 5%).

Higher Sen with lower Spe				Higher Spe with lower Sen			
Sen	1-Spe	J	CIB	Sen	1-Spe	J	CIB
0.950	0.450	0.500	0.298	0.550	0.050	0.500	0.421
0.950	0.350	0.600	0.360	0.650	0.050	0.600	0.494
0.950	0.250	0.700	0.432	0.750	0.050	0.700	0.564
0.950	0.150	0.800	0.523	0.850	0.050	0.800	0.632
0.950	0.050	0.900	0.699	0.950	0.050	0.900	0.699

For ROC analysis, the simulated sample size was 5000, and the results for the case–control study are shown in Table 3. The results showed that the optimum cut-off values of J and CIB were different when the incidence was 0.05.

**Table 3:** ROC analysis of J and CIB (incidence = 0.05) to determine the optimum cut-off value in a simulated dataset.

Model	Cut-off	Sen	1-Spe	J	CIB (incidence = 0.05)
Model A AUC = 0.701	145.0	0.07	0.01	0.06	0.141
	132.5	0.19	0.05	0.14	0.132
	125.5	0.30	0.10	0.20	0.149
	116.7	0.46	0.20	0.26	0.167
	**110.6**	0.58	0.30	0.28	**0.171**
	**105.5**	0.68	0.40	**0.28**	0.167
	100.2	0.77	0.50	0.27	0.161
Model B AUC = 0.814	144.2	0.17	0.01	0.16	0.295
	131.5	0.37	0.05	0.32	0.283
	124.9	0.49	0.10	0.39	0.283
	**116.0**	0.67	0.20	0.47	**0.299**
	**110.2**	0.77	0.30	**0.47**	0.286
	104.7	0.84	0.40	0.44	0.263
	99.7	0.89	0.50	0.39	0.232
Model C AUC = 0.922	148.0	0.35	0.01	0.34	0.477
	**133.0**	0.65	0.05	0.60	**0.494**
	126.1	0.76	0.10	0.66	0.466
	**116.9**	0.89	0.20	**0.69**	0.436
	110.8	0.93	0.30	0.63	0.383
	105.3	0.96	0.40	0.56	0.334
	99.9	0.98	0.50	0.48	0.286

Boldface: Optimum cut-off value.

**Figure 4:**
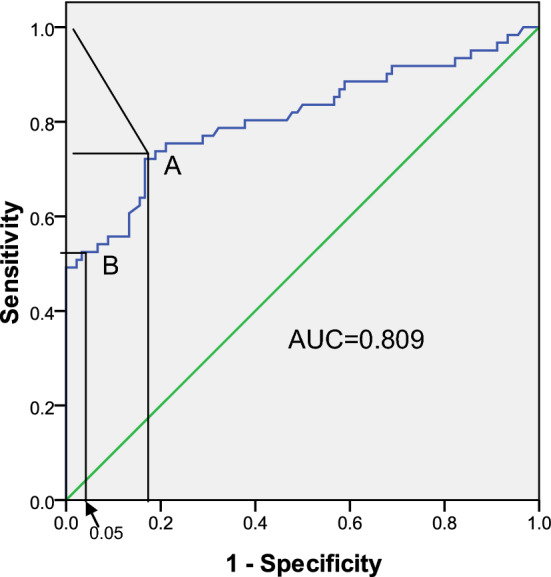
ROC analysis of tumor marker index (TMI) for the diagnosis and screening of gastric cancer. (**A**) optimum cut-off value for diagnosis (incidence = 0.50); (**B**) optimum cut-off value for screening (incidence = 0.05).

Actual data from our previous research were used for evaluating biomarker efficacy. In our previous research, TMI derived from serial tumor markers was found to be useful for the diagnosis of gastric cancer based on ROC analysis (Fig. 4 and Table 4). As shown in Fig. 4, the optimum cut-off values for diagnosis (incidence = 0.50) and for screening (incidence = 0.05) were different. The results indicate that if the cardinal number (value in the control group) is very small (and Spe is much higher), there could be an unsteady increase in CIB. Therefore, this frequency should be considered as 0.05 to calculate CIB, as shown in Table 4.

**Table 4:** Valuating the diagnostic and screening efficacy of tumor marker index (TMI) for gastric cancer with ROC analysis (incidence = 0.05).

Cut-off	Sen	1-Spe	J	CIB	Cut-off	Sen	1-Spe	J	CIB
0.765	0.787	0.322	0.465	0.281	0.950	0.672	0.167	0.505	0.330
0.770	0.770	0.311	0.459	0.279	0.976	0.639	0.167	0.472	0.309
0.772	0.770	0.300	0.470	0.286	0.990	0.639	0.156	0.483	0.319
0.774	0.770	0.289	0.481	0.294	0.995	0.623	0.156	0.467	0.309
0.776	0.754	0.289	0.465	0.284	1.507	0.607	0.133	0.474	0.322
0.785	0.754	0.278	0.476	0.292	2.031	0.590	0.133	0.457	0.311
0.795	0.754	0.267	0.487	0.300	2.060	0.557	0.133	0.424	0.289
0.799	0.754	0.256	0.498	0.308	2.076	0.557	0.122	0.435	0.301
0.811	0.754	0.244	0.510	0.317	2.080	0.557	0.111	0.446	0.315
0.822	0.754	0.233	0.521	0.325	2.113	0.557	0.100	0.457	0.329
0.823	0.754	0.222	0.532	0.334	2.153	0.557	0.089	0.468	0.345
0.834	0.754	0.211	0.543	0.343	2.174	0.541	0.089	0.452	0.334
0.860	0.738	0.211	0.527	0.333	2.200	0.541	0.078	0.463	0.352
0.878	0.738	0.200	0.538	0.342	2.223	0.541	0.067	0.474	0.373
0.882	0.738	0.189	0.549	0.351	2.237	0.525	0.067	0.458	0.362
0.887	0.721	0.189	0.532	0.341	2.280	0.525	0.056	0.469	0.387
0.891	0.721	0.178	0.543	0.351	**2.339**	0.525	0.044*	0.481	**0.403**
**0.897**	0.721	0.167	**0.554**	0.361	2.379	0.525	0.033*	0.492	0.403
0.910	0.705	0.167	0.538	0.351	2.406	0.508	0.033*	0.475	0.390
0.928	0.689	0.167	0.522	0.341	2.419	0.508	0.022*	0.486	0.390

*The cardinal number is less than 0.05; hence, this value should be assumed as 0.05 to calculate CIB; Boldface: Optimum cut-off value.

## Discussion

In the present study, we have proposed and evaluated an index for evaluating the diagnostic and screening efficacy of biomarkers for specific diseases. This index, CIB, is calculated using the consistency rate determined from case–control studies (J) and cohort studies (Crc). In fact, CIB comprehensively incorporates Sen, Spe, PPV, and NPV.

Our results show that when the incidence is 0.50, the J score is similar to CIB. As the subjects considered for diagnosis are usually symptomatic, the occurrence of a disease can be assumed to be a high-probability event for which the incidence can be set as 0.50. Therefore, for determining the diagnostic efficacy of a biomarker, J has similar power as CIB. In contrast, there is a significant difference between J and CIB in a low-probability event (probability = 0.05). As the subjects considered in screening for a disease are usually healthy and asymptomatic, the occurrence of a disease is assumed to be a low-probability event for which the incidence can be set as 0.05. Therefore, for determining the screening efficacy of a biomarker, J may not have as much power as CIB. Overall, our findings indicate that CIB may have potential for evaluating the screening efficacy of disease biomarkers.

For determining the screening efficacy based on CIB, the incidence (m) should be considered as 0.05 because test indicators usually include a 95% population interval as a reference range, with 5% of the population outside the normal reference range. The results showed that at an incidence of 0.05, ROC analysis of CIB showed an increase in the area under the curve. Thus, ROC analysis could be used to determine the cut-off values for screening purposes. The results indicated that higher Spe at a similar J value could indicate better power (and higher CIB), as shown in Table 2. Thus, CIB could increase unsteadily with J. Therefore, if the cardinal number (frequency in the control group) is very small (and Spe is much higher), this value should be assumed as 0.05 to calculate CIB.

Because the CIB range is typically 0–1, we propose that a CIB value of > 0.50 be considered to have clinical value^3^. However, diagnostic value is not necessarily equivalent to screening value, as shown in Table 3. Evaluation of biomarker efficacy using actual data from our previous also showed that TMI, which is derived from serial tumor markers, was more suitable for diagnosis than screening (Table 4). From analysis of the actual data, we also found that the J value from the case–control design was significantly larger than the CIB value for a low-probability event. This confirms the overestimation of J in low-probability events. Another example is the analysis of genetic associations (screening based on genetic markers), which has been successful in mapping genes, but is clinically inefficient because of inconsistent findings that have been partly attributed to overestimations in case–control studies. With the exception of Mendelian diseases, significant associations are difficult to detect because genetic diagnosis is usually used to screen healthy individuals for a disease, few genes have a CIB over 0.5, it might be misleading to pay attention only to the results for J from case–control studies. A statistical difference does not necessarily represent strong clinical effects, and diagnostic value does not always imply screening value.

It should be pointed out that to simplify the calculation, the incidence value in the present study was assumed to be 0.50 for diagnosis and 0.05 for screening. However, a more accurate estimation of CIB could be obtained based on the actual incidence of a disease. This is a line of investigation to pursue in the future.

In conclusion, CIB, which combines the consistency rates obtained from both case–control and cohort studies, could be more useful than J for determining the efficacy of a biomarker for screening purposes. It was also found that the efficacy of a biomarker could differ for diagnostic, screening, predictive, and prognostic purposes, and it would be better to evaluate the efficacy of biomarkers for specific systems or contexts.
